# Antepartum Depression and Anxiety Associated with Disability in African Women: Cross-Sectional Results from the CDS Study in Ghana and Côte d'Ivoire

**DOI:** 10.1371/journal.pone.0048396

**Published:** 2012-10-26

**Authors:** Carola Bindt, John Appiah-Poku, Marguerite Te Bonle, Stefanie Schoppen, Torsten Feldt, Claus Barkmann, Mathurin Koffi, Jana Baum, Samuel Blay Nguah, Harry Tagbor, Nan Guo, Eliezer N'Goran, Stephan Ehrhardt, for the International CDS Study Group

**Affiliations:** 1 Department of Child and Adolescent Psychiatry, University Medical Center Hamburg-Eppendorf, Hamburg, Germany; 2 Department of Behavioural Sciences, School of Medical Sciences, Kwame Nkrumah University of Science and Technology, Kumasi, Ghana; 3 Centre de Guidance Infantile, Institut National de Santé Publique, Abidjan, Côte d'Ivoire; 4 Clinical Research Unit, Bernhard Nocht Institute for Tropical Medicine, Hamburg, Germany; 5 URES Daloa, Population Genetics and Molecular Epidemiology of Infectious Diseases, Abobo-Adjamé University, Abidjan, Côte d'Ivoire; 6 Department of Child Health, Komfo Anokye Teaching Hospital, Kumasi, Ghana; 7 Department Community Health, School of Medical Sciences, Kwame Nkrumah University of Science and Technology, Kumasi, Ghana; 8 Department of Epidemiology, Johns Hopkins Bloomberg School of Public Health, Baltimore, Maryland, United States of America; 9 Research Unit of Parasitology and Parasite Ecology at UFR Biosciences, Université de Cocody, Abidjan, Côte d'Ivoire; University of Iowa Hospitals & Clinics, United States of America

## Abstract

**Background:**

Common mental disorders, particularly unipolar depressive disorders, rank among the top 5 with respect to the global burden of disease. As a major public health concern, antepartum depression and anxiety not only affects the individual woman, but also her offspring. Data on the prevalence of common mental disorders in pregnant women in sub-Saharan Africa are scarce. We provide results from Ghana and Côte d'Ivoire.

**Methods:**

We subsequently recruited and screened n = 1030 women in the third trimester of their pregnancy for depressed mood, general anxiety, and perceived disability using the Patient Health Questionnaire depression module (PHQ-9), the 7-item Anxiety Scale (GAD-7), and the World Health Organisation Disability Assessment Schedule II (WHO-DAS 2.0, 12-item version). In addition to estimates of means and prevalence, a hierarchical linear regression model was calculated to determine the influence of antepartum depression and anxiety on disability.

**Results:**

In Ghana, 26.6% of women showed substantially depressed mood. In Côte d'Ivoire, this figure was even higher (32.9%). Clear indications for a generalized anxiety disorder were observed in 11.4% and 17.4% of pregnant women, respectively. Comorbidity of both conditions was common, affecting about 7.7% of Ghanaian and 12.6% of Ivorian participants. Pregnant women in both countries reported a high degree of disability regarding everyday activity limitations and participation restrictions. Controlled for country and age, depression and anxiety accounted for 33% of variance in the disability score.

**Conclusions:**

Antepartum depression and anxiety were highly prevalent in our sample and contributed substantially to perceived disability. These serious threats to health must be further investigated and more data are needed to comprehensively quantify the problem in sub-Saharan Africa.

## Introduction

Almost a decade ago, the WHO focused on mental health, and with the slogan “no health without mental health”, placed efforts in this field at the top of its agenda [1]. Unipolar depressive disorders rank among the top 5 leading contributors to the global burden of disease [2]. Projections suggest that by 2020, depression may become the second leading cause of disease worldwide as measured by disability-adjusted life years. Yet, there is a lack of prioritisation of mental health in low- and middle-income countries (LMICs). In Africa, mental health is particularly neglected, although a high prevalence of common mental disorders (CMD), like unipolar depression and anxiety, is assumed [3], [4].

Women are known to be more vulnerable to CMD than men [5]. Depression and anxiety decisively contribute to the high burden of health risks faced by mothers and their offspring around birth in LMICs [6]. Data from high-income countries suggest that antepartum CMD disturb biosocial adaptation to pregnancy, and disables women with regard to everyday functioning, family and community life requirements [7]. Very little, however, is known about the consequences of antepartum CMD for African women. It can be speculated that disability related to CMD may be even more harmful in low-resource settings, where pregnancy does not imply relief from daily workload and economic responsibility for family needs. The concept of disability, as measured by the World Health Organization Disability Assessment Schedule II (WHO-DAS 2.0) has been developed to quantify self-reported activity limitations and participation restrictions [8]. This and other self-reported outcomes are rapidly gaining attention in research since they are clearly relevant and not subject to adjudication.

We hypothesized that CMD are highly prevalent in pregnant women in sub-Saharan Africa and substantially contribute to disability. In order to obtain a first estimate, we examined this hypothesis exploring data from the Child Development Study (CDS). CDS is a prospective, population-based, observational cohort study comprising mothers and their infants in Ghana and Côte d'Ivoire. Here, we present cross-sectional data on CMD and disability of women in their last trimester of pregnancy.

## Methods

### 1. Study design

The CDS study aims at assessing the impact of communicable and non-communicable diseases on infant development in Ghana and Côte d'Ivoire. Women in their last trimester of pregnancy were consecutively recruited in two large hospitals during antenatal care visits. After giving birth, the respective infants were enrolled in a birth cohort that is currently followed-up. We present cross-sectional data from antepartum mothers at recruitment.

### 2. Setting and Participants

The study was conducted in two health care institutions in Ghana and Côte d`Ivoire: The Komfo Anokye Teaching Hospital (KATH) in Kumasi, Ghana is accessible for an urban clientele, reflecting the economical and political stability of the country. The Abobo Community Hospital in Abidjan, Côte d'Ivoire is located in one of the most deprived areas of the city, serving an underprivileged population affected by civil war (2002–2004), ongoing political instability, and resumed fighting after presidential elections in November 2010. Both hospitals in their specific countries were chosen as study sites because of the good infrastructure, presence of well-established antenatal services and a sufficient number of births to fulfill recruitment goals.

Between March 2010 and February 2011, all women in their third trimester of pregnancy, based on gestational age, residing within a distance of ≤5 km around the hospitals, willing and able to provide informed consent were eligible for participation. The sample was restricted to women without severe obstetric risks factors since these may contribute to disability and adverse birth outcome. Exclusion criteria were: age under 18 years, multiple pregnancy and severe pregnancy complications, e.g. hypertension, hemorrhage, pre-eclampsia, and diabetes.

### 3. Procedures

After giving written informed consent, women were interviewed to obtain socio-demographic details (such as age and socioeconomic status) and information on obstetric factors. Mental health and disability assessments were performed by a team of two multilingual, resident psychology students, who were trained in the use of screening instruments (see below) by an expert psychiatrist. Constant supervision by an experienced team of national and international psychiatrists and psychologists assured quality of data and, if required, clinical intervention. All instruments were translated from English into the local language Twi in Ghana and back. For Côte d'Ivoire, the official translations into French, provided by the original authors, were used. Given the high illiteracy in both settings, questionnaires had to be adapted for interviewer administration. Medical data were obtained routinely in the two hospitals.

### 4. Ethics

This study was conducted in accordance with the ethical principles of the Declaration of Helsinki. It was approved by the ethical committee of the Kwame Nkrumah University of Science and Technology in Kumasi, Ghana (Ref: CHRPE/KNUST/KATH/01_06_08), the National Ethical Committee in Côte d'Ivoire (Ref: 4169/MHSP), and the respective committee of the Chamber of Physicians in Hamburg, Germany (Ref: PV3020). Generally, persons suffering from non-psychotic depression or anxiety are able to understand and consent to study requirements. Psychiatric disorders leading to severe cognitive impairment are rare, and found mainly in acute psychosis or dementia. In our study population of pregnant women drawn from the community, our psychological and psychiatric team did not encounter persons with a compromised capacity to fully understand the study participation rules.

### 5. Variables and Instruments

#### 5.1. Sociodemographic, anthropometric and basic medical data

A specifically designed questionnaire was completed by the pregnant women to assess socioeconomic status (SES). This questionnaire comprised 15 items on different aspects of region-specific issues of life standard such as education, occupation, and religion, but also on access to cooking gas, a flush toilet, and a freezer in the household. Weight and height were measured and recorded upon recruitment. A blood sample was obtained from every participant into EDTA (Ethylendiamin-Tetraacetat). A full blood count was done using a Coulter Counter.

#### 5.2. Depression

Depression was screened for with the Patient Health Questionnaire depression module (PHQ-9) [9]. The PHQ-9 refers to the past two weeks and assesses the presence and severity of the nine DSM-IV depression criteria, comprising emotional, cognitive, and functional somatic symptoms [10]. Response options generate a continuous score ranging from 0 (no symptoms) to 27 (all symptoms present nearly every day); scores 10–14 represent moderate and 15–27 moderately severe to severe depression symptoms. The PHQ-9 has been validated for use in primary care and in the general population in both high-income and low-income settings. A threshold score of ≥10 had a sensitivity of 88% and a specificity of 88% for major depression [11] and was used for case classification. The term “depression” does therefore not refer to a clinical diagnosis but to the result of a screening procedure in an epidemiological study with the above-mentioned properties. In rural postpartum Ghanaian women, the PHQ-9 proved superior to other common depression screening measures against a semi-structured clinical interview as reference standard [12]. The reliability of the raw score was Cronbachs α = .69 for the Ghanaian women and .64 for the women from Côte d'Ivoire.

#### 5.3. Anxiety

Anxiety was assessed using the GAD-7, a screening questionnaire for generalized anxiety disorder (GAD) according to seven DSM-IV symptoms [13]. The GAD-7 has a response set similar to the PHQ-9, comprising emotional and cognitive symptoms of anxiety during the past two weeks. Item scores range from 0 to 21, with 5–9 representing mild, 10–14 moderate, and 15–21 severe levels of anxiety. The threshold score of ≥10 has a sensitivity of 89% and a specificity of 82% for a generalized anxiety disorder and was used for case classification. The term “anxiety” does also not refer to a clinical diagnosis but to the result of a screening procedure in an epidemiological study with the above-mentioned properties. The GAD-7 has been validated in western primary care, but not in antepartum populations or in African settings, as research in antepartum anxiety is still in its early stages [14], [15]. In our study, the reliability of the raw score was Cronbachs α = .68 in both countries.

#### 5.4. Perceived Disability

Disability was assessed with the 12-item interviewer administered version of the World Health Organisation Disability Assessment Schedule II (WHO-DAS 2.0). This interview is based on the International Classification of Functioning, Disability and Health [8] and was designed to evaluate activity limitations and participation restrictions irrespective of medical diagnosis. Its items cover six domains of common daily life activities: understanding and communicating with the world; moving and getting around; self-care; getting along with people; life activities; and participation in society. In each item, respondents estimate the magnitude of their disability during the previous 30 days using a five-point scale. Scores for each question range from 0 (no difficulty) to 4 (extreme difficulty/cannot do), providing a range from 0 (no disability) to 48 (maximum disability) for the total score. We used the sum scoring method to produce a score for each participant. The WHO-DAS 2.0 was developed and field-tested cross-culturally, and found to be applicable both in clinical settings and in the general population [16], [17]. The short 12-item version correlates highly with the full 36-item version of the WHO-DAS 2.0, and proved to be a useful screening instrument [18], [19]. Cronbachs α in our samples was .79 and .76, respectively.

### 6. Statistical analyses

Initially, the data were analyzed by means of descriptive statistics and basic difference tests (e. g. Fisher's exact test, chi-square test, and ANOVA). Estimates of prevalence were complemented by symmetric 95% confidence intervals. The prediction of disability was conducted by a theory-driven sequential multiple linear regression analysis (blockwise forward). Country and age were included in the basic model based on our prior assumptions [20] and their statistically significant performance in the unadjusted models. Depression and anxiety were sequentially added in the second and final model. The adjusted R^2^ of each model is reported. Adjusted variables plots and residual plots were used to search for non-linear patterns, influential points and changing variance, and our data showed no violation of the linear assumption. Multi-collinearity was examined by calculating the variance inflation factor (VIF). With no VIF greater than 10 we found no indication of multi-collinearity. All tests were performed with a local two-tailed significance level of .05. Effect sizes were evaluated according to Cohen [21].

## Results

A total of n = 1030 mothers (299 from Ghana and 731 from Côte d'Ivoire) were consecutively recruited in the last trimester of their pregnancy. Less than 5% of eligible mothers refused to participate. In Ghana, n = 299 women and in Côte d'Ivoire n = 731 women were included. The reason for the higher quantity of mothers from Côte d'Ivoire is that study participants were lost for the cohort study due to confusion during the armed conflict and additional participants were recruited.

### 1. Sociodemographic data

**Figure 1:**
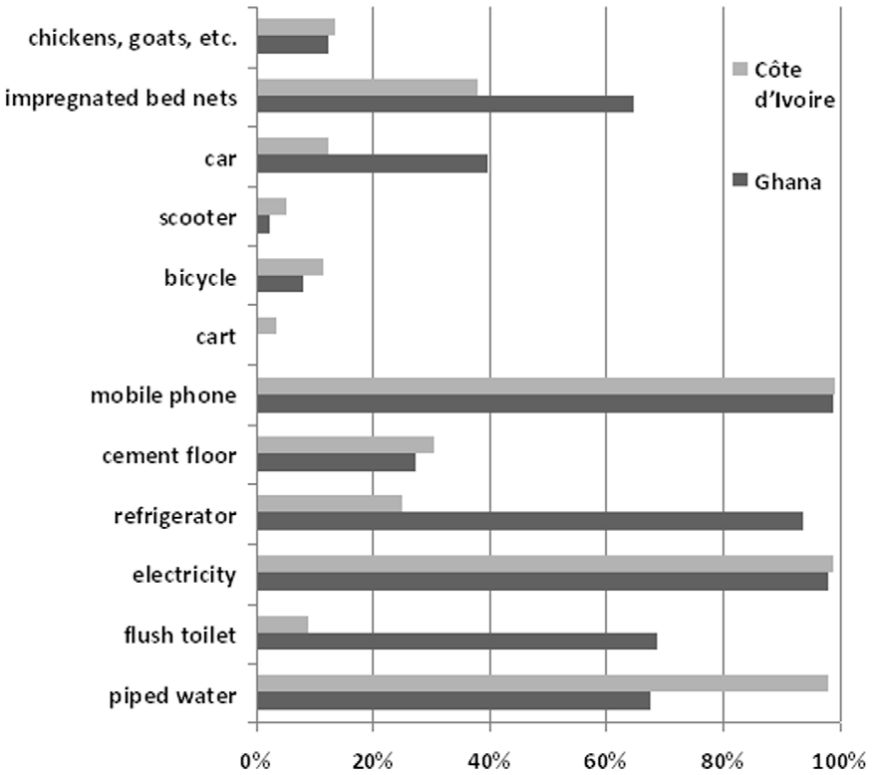
Household related life standard of pregnant women in Ghana and Côte d'Ivoire.

In Ghana and in Côte d'Ivoire, 93.3% (n = 279) and 56.5% (n = 409) of women were of Christian religion, respectively (others were mainly Islamic). No education or only primary education was reported by 38.6% (n = 115) of the women from Ghana and 66.3% (n = 480) of the women from Côte d'Ivoire. Figure 1 presents a simplified overview of the household-related life situation of the respondents in both countries. While there are basic differences between both countries (e.g., the availability of electricity), there are also similarities such as the availability of mobile phones. To explore the influence of SES on depression, anxiety and disability we constructed different SES variables. Some were associated with depression or anxiety but neither of these variables nor their combination was associated with disability.

### 2. Somatic data

Anthropometric and basic medical data of the study population are shown in table 1. Women were younger and weighed less despite being taller in Côte d'Ivoire as compared to Ghana. They also had lower mean hemoglobin (Hb) levels. However, most effect sizes were small. 33.3% (n = 92) of Ghanaian women reported some complications during previous pregnancies, as did 18.0% of the Ivorian women (n = 129; Fishers exact test: p<.0001). Caesarean sections in previous pregnancies were more common in Ghana as compared to Côte d'Ivoire: 19.2% (n = 54) and 6.5% (n = 45; Fishers exact test: p<.0001) of the women, respectively, had one or more Caesarean sections. Non-severe pregnancy complications requiring medical intervention during the time of investigation (e.g., pelvic infections or cervical insufficiency) were observed in 2.0% (n = 6) of Ghanaian and 4.3% (n = 30; Fishers exact test: p = .095) of Ivorian women.

**Table 1:** Anthropometric and basic medical data of the sample, split by country.

	Ghana (N = 299)			Côte d'Ivoire (N = 731)			d
	M_1_	SD_1_	n_1_	M_2_	SD_2_	n_2_	
Age (years)	29.6	4.88	299	28.4	5.85	731	0.20*
Weight (kg)	74.0	11.64	298	68.3	12.16	727	0.47***
Height (cm)	159.3	5.98	299	162.3	5.91	714	−0.50***
Hemoglobin level (g/dl)	11.2	1.11	294	10.7	1.30	213	0.38***
Previous pregnancies	2.9	1.90	292	3.1	2.00	655	−0.12ns

Notes. d  =  Hedges g  =  (M_1_ – M_2_)/SD_pooled_, small: d≤.20, medium: d≤.50, large: d≤.80; ns: p>.050, *: p≤.050, **: p≤.010, ***: p≤.001.

### 3. Depression, anxiety and disability

The distribution of depression, anxiety, and disability among study participants scored by the PHQ-9, GAD-7, and WHO-DAS 2.0 stratified by country is displayed in table 2. The distribution of scores for depressive symptoms was slightly skewed to the right for both groups, and mean values were higher in Ivorian women. Frequencies in the different cut-off groups differed somewhat between Ghanaian and Ivorian women. When the cut-off ≥10 PHQ-9 raw score was used for case identification, 26.6% (95%-CI  = 21.7; 31.8) of Ghanaian and 32.9% (95%-CI  = 29.5; 36.4) of Ivorian women had scores indicating a major depression according to DSM-IV criteria. A similar pattern of symptom distribution existed for anxiety, only somewhat more pronounced than for depression. The corresponding cut-off ≥10 GAD-7 raw score resulted in 11.4% (95%-CI  = 7.8; 15.0) and 17.4% (95%-CI  = 14.6; 20.2) Ghanaian and Ivorian pregnant women, respectively, with scores suggestive of a generalized anxiety disorder according to DSM-IV. Criteria for both major depression and generalized anxiety disorder were simultaneously fulfilled by 7.7% (95%-CI  = 4.7; 10.7) of women from Ghana and 12.6% (95%-CI  = 10.2; 15.0) of women from Côte d'Ivoire. Mean levels of disability were also high. Differences between the countries were small but reached statistical significance. A sensitivity analysis of the Ivorian sub-sample pre- and post-conflict revealed that mean disability scores did not differ significantly. Pre- and post-conflict PHQ-9 (M = 8.8, SD  = 4.48 and M = 7.4, SD  = 4.49, respectively) and GAD-7 (M = 6.8, SD  = 4.33 and M = 5.4, SD  = 3.69, respectively) scores differed significantly (p<.001) but differences were judged to be of little clinical relevance.

**Table 2:** Depression, anxiety and disability in pregnant women in Ghana and Côte d'Ivoire.

	Ghana		Côte d'Ivoire		d
	(N = 299)		(N = 731)		
Depression (PHQ-9)					
Total raw score (M, SD)	7.4	4.65	7.8	4.64	−.10*
None (0–4; %, n)	34.1	102	26.9	194	
Mild (5–9; %, n)	39.1	117	40.1	289	
Moderate (10–14; %, n)	17.1	51	23.3	168	
Moderately severe (15–19; %, n)	8.4	25	8.5	61	
Severe (20–27; %, n)	1.3	4	1.1	8	
Anxiety (GAD-7)					
Total raw score (M, SD)	4.7	3.83	5.8	3.95	−.27***
None (0–4; %, n)	56.5	169	44.1	317	
Mild (5–9; %, n)	32.1	96	38.5	277	
Moderate (10–14; %, n)	9.4	28	14.2	102	
Severe (15–21; %, n)	2.0	6	3.2	23	
Disability (WHO-DAS 2.0)					
Total raw score (M, SD)	13.2	6.66	11.8	7.32	+0.19**

Notes. PHQ-9 total raw score ranges from 0–27; GAD-7 total raw score ranges from 0–21; WHO-DAS 2.0 ranges from 0–48, no cut-offs established yet; d  =  Hedges g  =  (M1 – M2)/SD_pooled_, small: d≤.20, medium: d≤.50, large: d≤.80; ns: p>.050, *: p≤.050, **: p≤.010, ***: p≤.001.

### 4. Prediction of disability

SES, complications during the ongoing pregnancy and a history of Caesarean sections were not associated with disability. Table 3 shows the average disability score for people with no depression and anxiety, depression only, anxiety only and comorbidity stratified by age group. Late pregnancy seems to add substantially to “background disability” in a subsample of n = 658 non-depressed and non-anxious women, even more so with increasing age. Table 4 shows the results of the sequential models. In the basic model, country and age explained 2% of the variance in the disability score. In model 1, the explained variance increased to 32% after the inclusion of depression. In the final model, country, age, depression, and anxiety explained the 33% of the variance. Our data suggest depression, anxiety and, particularly, comorbidity to be strong drivers of higher disability scores.

**Table 3:** WHO-DAS 2.0 raw scores. Contribution of depression, anxiety and both to disability, stratified by age group.

	No depression, no anxiety		Depression only		Anxiety only		Comorbidity	
Age group	N	Mean (SD)	N	Mean (SD)	N	Mean (SD)	N	Mean (SD)
18–24	158	8.6 (5.80)	57	14.2 (6.69)	10	12.8 (6.60)	26	17.0 (6.45)
25–34	397	10.4 (5.94)	109	16.5 (6.47)	27	12.6 (4.27)	62	19.9 (6.65)
35–46	102	10.7 (6.73)	35	16.6 (6.95)	7	17.6 (5.74)	27	20.5 (5.77)
Total	658	10.0 (6.08)	201	15.9 (6.67)	44	13.4 (5.30)	115	19.4 (6.49)

Notes. No depression, no anxiety: PHQ-9 and GAD-7 scores <10; depression only, PHQ-9 score ≥10 and GAD-7 score <10; anxiety only, PHQ-9 score <10 and GAD-7 score ≥10; comorbidity, PHQ-9 and GAD-7 scores ≥10.

**Table 4:** Sequential regression of depression and anxiety on disability of pregnant woman in Ghana and Côte d'Ivoire.

	Model 0		Model 1		Model 2	
	B	95%-CI	B	95%-CI	B	95%-CI
Intercept	5.77	3.30; 8.24	0.08	−2.06; 2.22	−0.85	−3.00; 1.29
Control variables						
Country (Ghana, Côte d'Ivoire)	1.11*	0.35; 2.32	1.36***	0.56; 2.15	1.60***	0.81; 2.40
Age	0.17***	0.10; 0.25	0.14***	0.08; 0.21	0.15***	0.08; 0.21
Independent variables						
Anxiety (GAD-7)					0.29***	0.18; 0.40
Depression (PHQ-9)			0.83***	0.75; 0.91	0.70***	0.61; 0.79
Adjusted R^2^	0.02		0.32		0.33	
R^2^ Change from model 0			0.30		0.31	

Notes. PHQ-9 score ranges from 0-27; GAD-7 score ranges from 0–21; WHO-DAS 2.0 score ranges from 0–48, * p≤.05; ** p≤.01; *** p≤.001; N_model 0_  = 947; N_model 1_  =  N_model 2_  = 936.

## Discussion

The results of this study suggest high prevalences of depression, anxiety, or both, in a sample of urban West-African women during the last trimester of their pregnancy. Both conditions contribute substantially to disability. In Ghana, one fourth of the women scored within the range of clinically relevant depression, while in Côte d'Ivoire, one third of the studied participants did. Although in the upper limits of the spectrum, our results are in line with the patchy data available from Africa. Sawyer et al. (2009) found a mean prevalence of antepartum depression of 11.3%, summarizing five studies from three African countries [22]. Only recently, 39% of pregnant women in a South African sample were classified as depressed [23]. Antepartum anxiety was assessed in two studies from Nigeria. While Esimai et al. [24] reported 5.8%, Adewuya et al. [25] found 39%. Our findings lend evidence to the assumption that CMD during gestation is not a culture-bound Western phenomenon [22].

The proportion of depressed women in both countries substantially exceeded the respective figures for anxiety. It is well recognized that the clinical presentation of depression and anxiety partly overlap, complicating discrimination between the two [26]. Substantial comorbidity between antenatal anxiety and depression has been found, accounting for up to 50% of affected women [15]. In our study, we chose the PHQ-9 and GAD-7 as screening instruments, which are constructed alike with respect to DSM-IV diagnostic criteria. The PHQ-9 explicitly relinquishes anxiety symptoms from its scale to address depression only, which allows exploration of two distinguished symptom sets in our subjects with no overlap between items. In Ghana, 7.7% women had both PHQ-9 and GAD-7 scores ≥10, indicative of comorbidity of depression and anxiety. In Côte d'Ivoire, this figure was 12.6%. We conclude that substantial comorbidity exists in our sample.

The significant differences in CMD prevalence between Ghanaian and Ivorian women could not be explained by differences in socioeconomic status, although Ivorian women appeared to be more disadvantaged. Thus, we can only speculate about the causes of this finding. One difference between both countries is the political situation. While Ghana remained stable for almost two decades, Côte d'Ivoire was exposed to severe political unrest and warfare between 2002 and 2007, and again 6 months after we started recruitment. This ongoing threat may have led to increased mental distress, reducing the impact of socioeconomic risks. Pervasive high prevalences of CMD have been widely documented in people afflicted by war [27].

The WHO defines disability as “the negative aspects of the interaction between an individual and that individuaĺs contextual factors” [8], shifting focus from cause to impact of any health impairment. We explored whether psychological distress in pregnant African women was related to disability. The WHO-DAS 2.0 aims at identifying individual activity and participation restrictions. It may be used to compare group differences within countries but care is recommended when interpreting cross-national differences [16], [17]. There is no agreed cut-point for identifying persons with substantial disability, and norms for Ghanaian or Ivorian pregnant women have not yet been established. Nonetheless, the multinational World Mental Health Survey reported that most respondents in large population samples had zero scores in each domain of the WHO-DAS 2.0 scale [17]. In an Australian population based survey, subjects scoring >10 on the 12-item version scale (scores 0–48) were in the top 10% of the population distribution of scores, and were likely to have clinically significant disability. The mean WHO-DAS 2.0 score for people with a common mental disorder was 6.3 (SD  = 7.1). In a subgroup with a mental but no physical disorder the disability score was M = 4.2 (SD  = 5.2) [20].

Late pregnancy itself may be a burdensome condition, and we expected some degree of disability in the third trimester. Women who were classified as not depressed and not anxious (n = 658) had a mean score of 10.0 (SD  = 6.08) on the WHO-DAS 2.0 disability scale. Mean score increases in women with depression, anxiety, or both were about 6, 3.5, and 9.5, respectively. Older women appeared to have higher disability score increases as compared to younger women. Increasing disability with age has been reported before [20]. We can only speculate that physical strain due to late pregnancy may have accounted for the high “background disability” in our sample. Increases above this background disability result in high disability scores in women with CMD, but these increases, though not the absolute values are somewhat in line with the Australian data [20]. Comorbidity of depression and anxiety, on average, doubled disability scores.

Depression and anxiety were clearly associated with high disability scores. Affected women did not only experience psychological distress, but also felt substantially disabled by their symptoms. Global data demonstrating depression to be a leading cause of disability [28] are reflected in our sample.

Worldwide, Africa has the highest proportion of people living in extreme poverty, which highly correlates with CMD [29]. We found some indication of SES being associated with depression and anxiety but effect sizes were small. This may indicate a relative socio-economic homogeneity of our sample. SES per se did not contribute to disability. A similar pattern has been reported in a different population from Korea [30].

Severe pregnancy complications are common in sub-Saharan Africa, and according to a recent meta-analysis, the incidence/prevalence ratio and case-fatality ratio for maternal near misses ranged from 1.1%–10.1% and 3.1%–37.4%, respectively [31]. In our study sites, the general percentage of women undergoing Caesarean sections was around 25% in both hospitals (S.B. Nguah, personal communication). Thus, the number of complications during previous pregnancies in our sample appears to reflect collective norm. Somewhat unexpected, there was no association between physical health factors (e.g., weight, blood pressure, hemoglobin level) and CMD or disability in our subjects. Pregnancy complications not leading to study exclusion and Caesarean sections in previous pregnancies were also not associated with disability. The strongest predictor of disability was depression.

We conclude that CMD, primarily depression, strongly impact on disability during pregnancy in our study population. This finding is worrying as are the high prevalences of antepartum CMD. CMD during pregnancy predicts maternal CMD throughout the first year postpartum [32], which in turn may impact on duration of breastfeeding, infant growth, morbidity, and child cognitive and behavioural development [33], [34], [35], [36]. CMD not only affect the woman, but also her unborn child. Various studies report negative effects of maternal depression and anxiety on the course of pregnancy and birth outcome due to poor antenatal care, complication of labour, preterm delivery, fetal growth restriction, and low Apgar scores [37], which are more pronounced among deprived social groups and in poor countries [38]. Consequences for the course of pregnancy, birth outcome and maternal and child health in our cohort have yet to be investigated.

### Strengths and limitations

This is the first study to examine depression, anxiety, disability and their correlates using screening instruments in a large sample of Ghanaian and Ivorian pregnant women. The cross-sectional nature of the study does not allow for causal inference. The sample was systematically recruited, refusal rates were low, and the instruments were applied by local mental health professionals. Yet, information bias may occur when using imperfect tests like the PHQ-9 and the GAD-7. Our main outcome the WHO-DAS 2.0, however, is self-reported. The structure of the samples, being pregnant women who attended hospital prenatal care, raises the possibility of selection bias. Our sample also appears to be rather homogeneous with regard to SES.

A difficulty faced when measuring antenatal depression or anxiety is that somatic symptoms related to pregnancy itself may lead to misclassification. Yet, our sample was restricted to women without severe pregnancy complications. The GAD-7 explicitly removes somatic symptoms, while the PHQ-9 emphasizes on emotional and cognitive symptoms, but assesses functional somatic symptoms, which have been found typical presentations of depression in African people [39].

Established measures to screen for anxiety in antepartum populations do not exist [15]. Because anxiety is probably common but rarely investigated in Africa [40], we decided to use the GAD-7, which proved suitable in primary care settings. The effect of the armed conflict in Côte d'Ivoire during the study period is difficult to assess. Post-conflict mean raw sores for the PHQ-9, GAD-7, and WHODAS 2.0 were slightly lower as compared to pre-conflict scores. Possibly, societal conflicts that precede armed conflicts affect a population long-term and this is reflected by our measures of depression, anxiety and disability.

### Implications for further research

Intensive research and clinical efforts need to be directed towards recognizing and understanding antepartum mental distress and disability. Paving the way to develop effective interventions that are suitable for integration into primary healthcare in LMICs is paramount.
